# Immune and nonimmune mechanisms mediate the mental stress-induced tumor growth in a xenograft model of breast cancer

**DOI:** 10.1038/s41419-021-04280-9

**Published:** 2021-10-23

**Authors:** Wenjing Ma, Pengfei Liu, Jie Zheng, Jinhui Lü, Qian Zhao, Danni Li, Yuefan Guo, Lu Qian, Qiong Wang, Xinman Miao, Zuoren Yu

**Affiliations:** 1 Research Center for Translational Medicine, Shanghai East Hospital, Tongji University School of Medicine, Shanghai, China; 2 School of Life Sciences and Technology, Tongji University, Shanghai, China; 3 Jinzhou Medical University, School of Basic Medical Sciences, Jinzhou, Liaoning Province China

**Keywords:** Breast cancer, Disease model, miRNAs

## Abstract

Excess mental stress may harm health, and even accelerate cancer initiation and progression. One fourth of breast cancer patients suffer mental stress including anxiety, sadness, or depression, which negatively affect prognosis and survival. However, the regulatory mechanism is yet to be determined. Herein, we applied unpredictable stress stimuli to the breast tumor-bearing mice to establish a xenograft model of breast cancer suffering mental stress, followed by behavioral tests, tumor growth tracking, immune analysis, miRNA screening, and tumor cell proliferation analysis as well. As a result, increased stress hormone levels in serum, decreased percentage of T and NK cells in both blood and tumor samples and accelerated tumor growth in vivo were observed in the mice exposed to mental stress. Promoted cell proliferation was observed in both primary tumor cells derived from the stressed mice and 4T1 breast cancer cells treated with stress hormone corticosterone. In addition, a subset of miRNAs including miR-326, 346, 493, 595, 615, and 665 were identified through a miRNA screening with downregulation in tumors of the stressed mice. CCND1 was identified as a common target gene of miR-346 and miR-493, the top two most significantly downregulated miRNAs by stress exposure. The stress-miRNA-CCND1 signaling regulation of the tumor cell proliferation was further validated in 4T1 cells treated with corticosterone in vitro. GO terms and KEGG pathways analyses on the target genes of miR-346 and miR-493 revealed their involvement in the regulation of human cancer and neuron system, indicating the importance of non-coding genome in mediating the mental stress-induced cancer regulation. In conclusion, this study not only explored immune and nonimmune mechanisms through which mental stress exposure contributes to tumor growth in breast cancer, but also suggested a new therapeutic strategy for cancer patients suffering mental stress.

## Introduction

Mental stress, as a natural part of life, is expressed by everyone from time to time. Emerging evidence has revealed the health risks of long-term and/or excessive stress suffering by any individuals. Although immediate or short-term stress may be beneficial to alert us to maintain the body’s homeostasis responding to serious situations by modifying blood pressure, heart rate, endocrine secretion and neuron activity [1–3], chronic stress exposure frequently leads to health problems including mental anxiety, sadness, depression, and physical headaches, sleeplessness, cardiovascular diseases, and even cancer initiation and progression [4–7]. In recent years, increasing studies clearly showed an association between long-term mental stress and tumorigenic risk [6, 8]. A meta-analysis indicated that individuals suffering excessive mental stress had shortened telomeres, increased pro-inflammatory cytokines, impaired immune function and imbalanced hormone levels, all of which are important factors regulating human cancers [8, 9]. A 4.72-fold increase of breast cancer risk was demonstrated for those women exposed to mental stress from bereavement, illness, or divorce. Moreover, those breast cancer patients suffering excessive anxiety or worrying about the illness showed a poorer prognosis [10, 11].

Two main mechanisms including immune and nonimmune mechanisms are believed to mediate the cancer regulation by mental stress. Hypothalamic-pituitary-adrenal (HPA) axis and sympathetic nervous system (SNS) activation have been shown to be direct target responding to mental stress exposure [7]. Subsequent release of stress hormones, such as catecholamines not only reduce the amount and activity of immune cells, but also inhibit cytotoxic T lymphocytes (CTL)-mediated immune response, thereby decreasing the anti-tumor immune responses [12]. Meanwhile, stress hormones have ability to promote the expression of oncogenes in cancer cells, thereby promoting the cellular proliferation, invasion, and migration [13, 14]. For example, stress-induced epinephrine secretion activated lactate dehydrogenase A (LDHA) to promote the myc-slug signaling, thereby enhancing the development of breast cancer stem-like traits [13].

MicroRNAs (miRNAs) play an important role in the post-transcriptional control of gene expression, regulating biological processes [15] and cancer development and progression [16, 17]. Stress exposure can directly regulate the expression of cancer-related mRNAs and miRNAs through nonimmune mechanisms. One of our recent studies for the first time reported the attenuated miRNA-target interaction by starvation stress stimulation in breast cancer cells [18]. Du et al. reported activation of miR-337-3p/STAT3 axis by chronic stress contributed to the promoted metastasis in breast cancer [19]. However, the non-coding genome involvement in the mental stress-induced tumor growth in breast cancer remains unclear.

Herein, we applied unpredictable stress stimuli to the breast tumor-bearing BALB/c mice for two weeks to establish a breast cancer model suffering mental stress, followed by the behavioral tests, tumor-growing tracking, immune system analysis, miRNA screening and cell proliferation analysis. Multiple approaches of tests demonstrated depressive-like behavior of the mice under mental stress condition, which was associated with promoted tumor growth, increased secretion of corticosterone (CORT) and decreased percentage of immune cells in vivo. The cell proliferation and the cell cycle analysis were performed in vitro with primary tumor cells and 4T1 breast cancer cells treated with CORT. In addition, a miRNA screening analysis identified six miRNAs with downregulation in the tumors of the mice exposed to mental stress, which were further validated in the CORT-treated 4T1 cells. GO and KEGG analyses indicated the involvement of those miRNAs in regulating human cancer and neuron system. A key regulator of the cell cycle, CCND1 was identified as a potential target gene of the top-two most significantly deregulated miRNAs by mental stress. The stress-miRNA-CCND1 signaling pathway might be responsible for the stress-induced breast tumor growth as a novel nonimmune mechanism.

## Results

### Behavioral disorders induced by mental stress exposure in the mammary gland tumor-bearing mice model

**Fig. 1:**
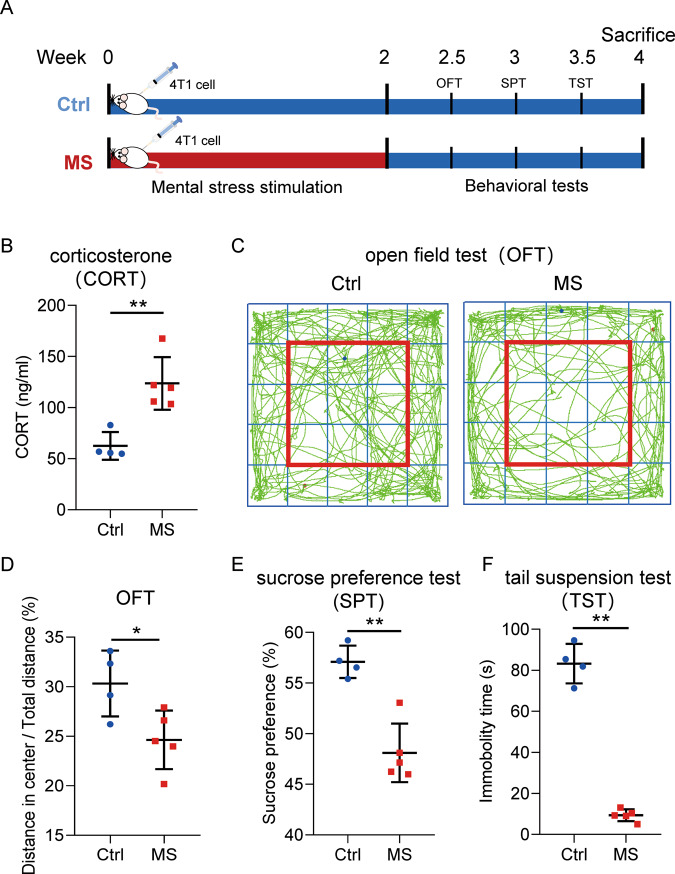
Establishment of a xenograft model of breast cancer suffering mental stress. **A** Schematic representation of the work flow to develop the model. **B** Increased levels of corticosterone (CORT) in the serum of the mice treated with mental stress. **C**, **D** Behavioral test of the mice by open field test (OFT). **E** Behavioral test of the mice by sucrose preference test (SPT). **F** Behavioral test of the mice by tail suspension test (TST). Ctrl: control; MS: mental stress. Data are presented as the mean ± SEM (*n* = 4 for Ctrl group, *n* = 5 for MS group). *p < 0.05, **p < 0.01.

To determine the influence of mental stress exposure to tumor growth in patients with breast cancer, a mammary gland tumor-bearing mice model was developed via 4T1 cell transplantation. A two-week exposure of unpredictable stress stimuli was given to the mice in the MS group, followed by three kinds of behavioral tests as shown in Fig. 1A. In order to confirm the effect of mental stress exposure, CORT, a major stress hormone in rodents, was measured in serum of the mice. In consistent with the literature [20], the serum level of CORT significantly increased after mental stress stimulation from ~50 ng/ml (Ctrl group) to ~100–150 ng/ml (MS group) (Fig. 1B). Behavioral changes induced by mental stress exposure to mice were determined, including decreased exploratory behavior and general activity (tested by Open field test (OFT), Fig. 1C, D), anhedonia natural preference for sweets (tested by sucrose preference test (SPT), Fig. 1E), and depressive-like behavior (tested by tail suspension test (TST), Fig. 1F).

### Mental stress exposure promoted tumor growth in vivo

**Fig. 2:**
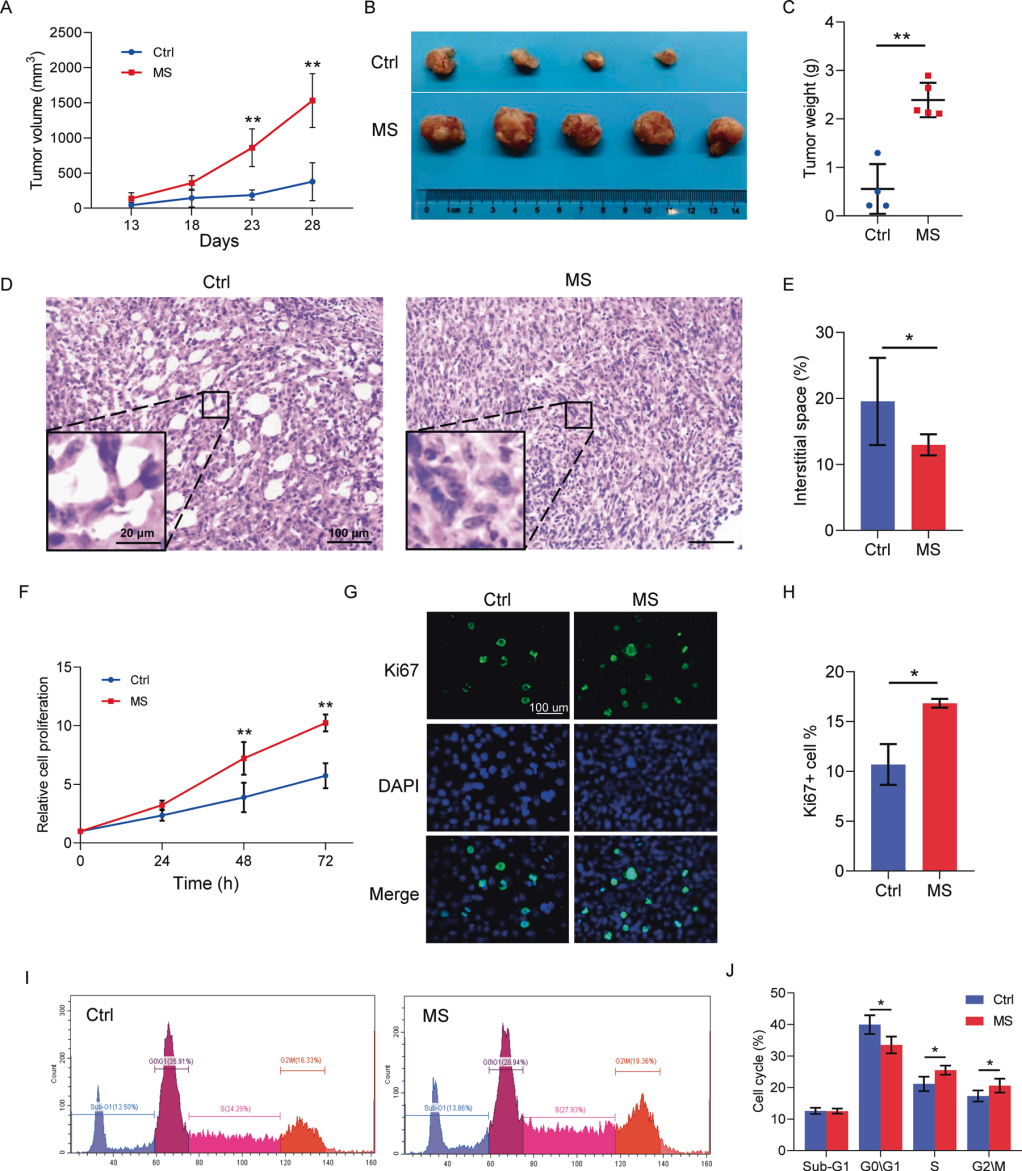
Mental stress exposure promoted tumor growth in vivo. **A** Tumor growth curve of the mice in MS and Ctrl groups. **B** Images of the mammary gland tumors separated from the mice at day 28 after the cancer cell transplantation. **C** Weight of the tumors from both groups of mice. **D** H&E staining of the paraffin-embedded tumor tissue sections indicating the increased tumor density after MS exposure to the mice. **E** Quantitative analysis of the tissue density in (**D**). **F** Cell proliferation analysis of the primary tumor cells by CCK8 assay. **G** Cell proliferation analysis of the primary tumor cells by Ki67 staining. **H** Quantitative analysis of the Ki67+ cells in (**G**). **I** The cell cycle analysis of the primary tumor cells by propidium iodide DNA staining and flow cytometry. **J** Quantitative analysis of the cells at different phases in (**I**). Data are presented as the mean ± SEM (in **A** and **C**, *n* = 4 for Ctrl group, *n* = 5 for MS group; in (**E**), (**F**), (**H**) and (**J**), *n* = 3 for both groups). *p < 0.05, **p < 0.01.

The influence of mental stress exposure on tumor progression of the mammary gland tumor-bearing mice was determined according to the tumor growth curve and tumor weight. As indicated in Fig. 2A–C, mental stress exposure dramatically promoted tumor growth in vivo, compared to the control mice without any stress stimulation. In addition, body weight of the mice in the MS group showed a reduced trend from the second week of stress exposure to the last day of week 4 when the experiments ended (Supplemental Fig. S1). Interestingly, it is the first time that we observed a higher level of tissue density in the mammary gland tumors of the mice in the MS group through hematoxylin and eosin (H&E) staining analysis (Fig. 2D, E). In view of the positive correlation between the level of breast density and risk of breast cancer in women, stress-induced breast tumor density might be an indication of promoted degradation of the regular extracellular matrix (ECM) and/or deposition of a tumor-specific ECM in the tumor microenvironment [21]. As well reviewed by Ni et al., ECM is important for the tumor-stroma interactions within tumor microenvironment [22].

In order to further validated the stress-induced tumor growth, primary tumor cells were isolated from the tumor tissues, and applied for cell proliferation assays. In consistent with the induced tumor growth in vivo, primary tumor cells from the stressed mice showed higher level of cell proliferation rate (Fig. 2F), higher proportion of Ki67+ tumor cells (Fig. 2G, H), and accelerated G1/S transition of the cell cycle (Fig. 2I, J), compared to primary tumor cells from the control mice.

### Mental stress exposure inhibited immune system in both blood and tumors

**Fig. 3:**
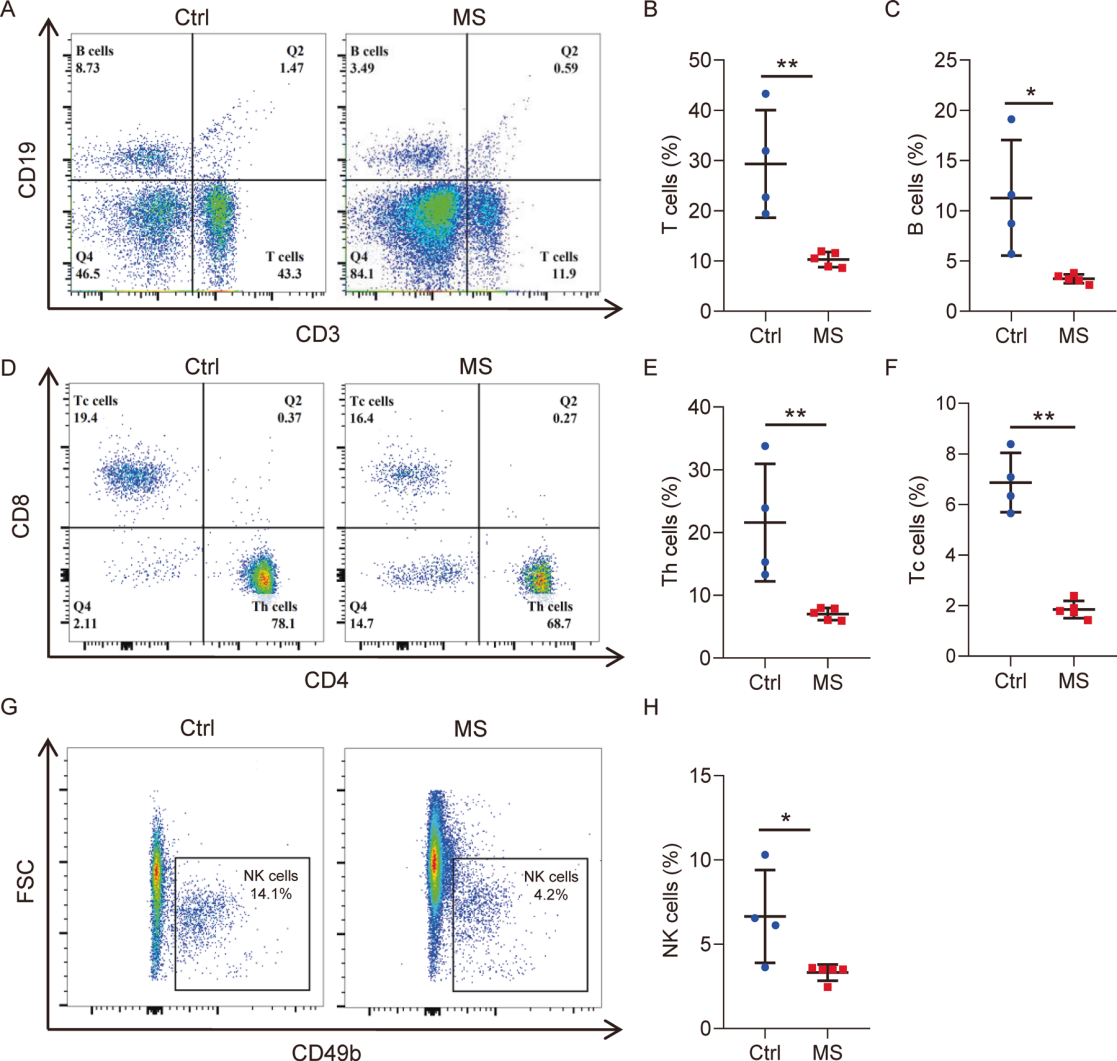
Mental stress exposure suppressed immune cells in peripheral blood of mice. **A** Flow cytometric analysis of immune cells in peripheral blood by staining of surface markers CD3 and CD19. **B** Quantitative analysis of T cells (CD3+ CD19−) in (**A**). **C** Quantitative analysis of B cells (CD3− CD19+) in (**A**). **D** Flow cytometric analysis of T cells in peripheral blood by staining of surface markers CD4 and CD8. **E** Quantitative analysis of Th cells (CD4+ CD8−) in (**D**). **F** Quantitative analysis of Tc cells (CD4− CD8+) in (**D**). **G** Flow cytometric analysis of natural killer (NK) cells in peripheral blood by staining CD49b. **H** Quantitative analysis of NK cells (CD49b+ CD3− CD19−) in (**G**). Data are presented as the mean ± SEM (*n* = 4 for Ctrl group, *n* = 5 for MS group). *p < 0.05, **p < 0.01.

To determine the immune mechanisms mediating the stress-induced phenotypes in the tumor-bearing mice, peripheral blood of the mice was applied for FACS analysis of immune cells. As shown in Fig. 3A–C, the percentage of both T cells (CD3+ CD19− cell type) and B cells (CD3− CD19+ cell type) dramatically decreased in the stressed mice. T cells in blood were further analyzed using surface markers CD4 and CD8, showing a reduction of both Th and Tc cells (Fig. 3D–F) by mental stress exposure. In addition, CD49b analysis demonstrated a reduction of natural killer (NK) cells in the blood of the stressed mice (Fig. 3G, H). As a result, stress exposure to the mammary gland tumor-bearing mice significantly suppressed T, B and NK lymphocytes in peripheral blood.

**Fig. 4:**
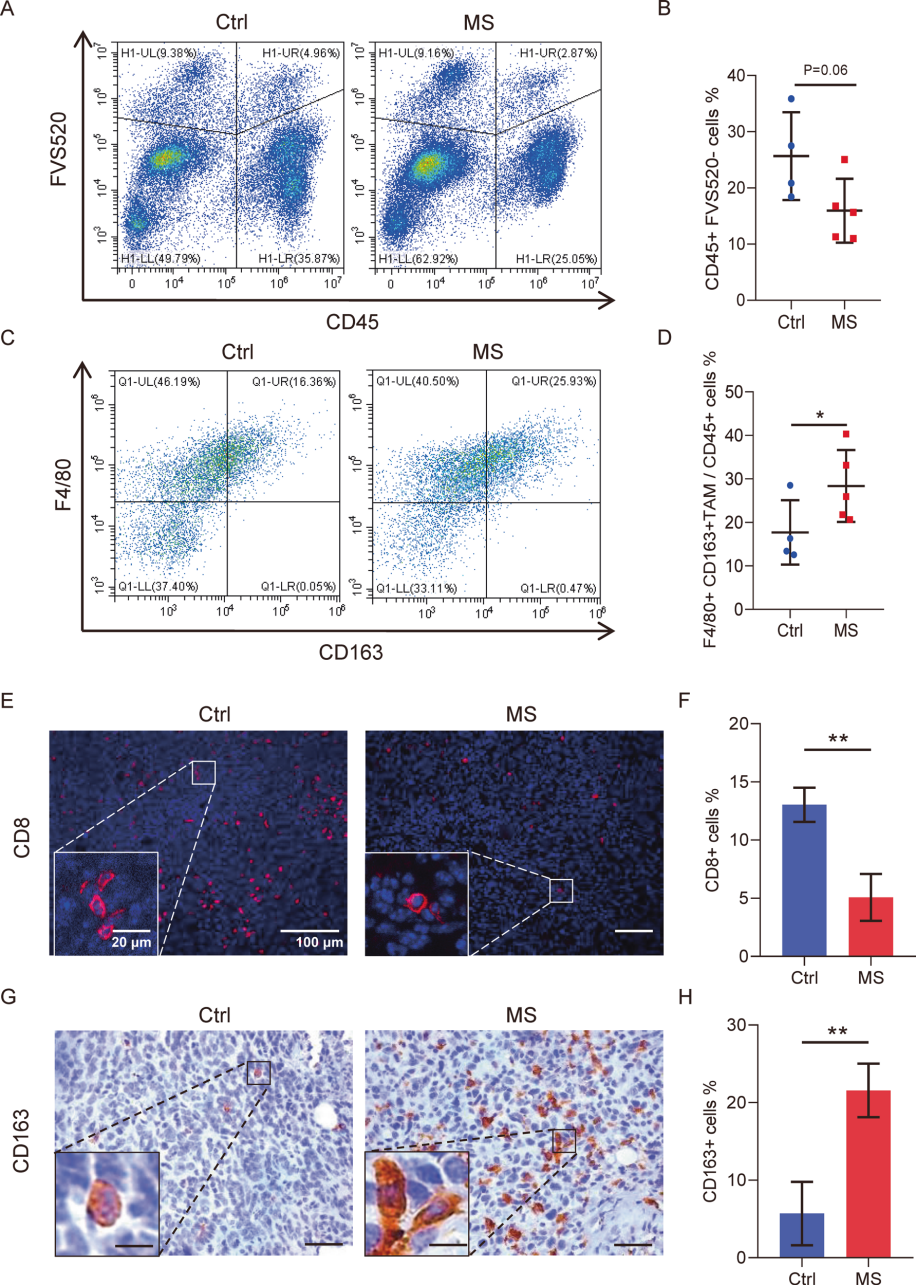
Mental stress exposure suppressed immune system in tumor tissues of mice. **A** Flow cytometric analysis of immune cells in the primary cells from tumor tissues by staining of surface markers CD45. FVS520 staining was used to distinguish living cells. **B** Quantitative analysis of CD45+ immune cells in (**A**). **C** Flow cytometric analysis of tumor-associated macrophages (TAM) in the primary cells from tumor tissues by staining of F4/80 and CD163. **D** Quantitative analysis of TAM cells (F4/80+ CD163+) in (**C**). **E** CD8+ T cell analysis in the tumor tissues by immunofluorescence (IF) staining on the paraffin sections. **F** Quantitative analysis of CD8+ T cells in (**E**). **G** CD163+ TAM analysis in the tumor tissues by immunohistochemistry (IHC) staining on the paraffin sections. **H** Quantitative analysis of CD163+ TAM in (**G**). Data are presented as the mean ± SEM (*n* = 4 for Ctrl group, *n* = 5 for MS group). *p < 0.05, **p < 0.01.

Moreover, immune cells in the tumor microenvironment of the mice were analyzed as well. CD45, as the leukocyte common antigen, was investigated in the tumors. CD45 was also expressed in the tumor-infiltrating T lymphocytes (TILs) cells which have been shown to associate with clinical outcomes and overall survival in patients with solid tumors [23]. As shown in Fig. 4A, B, CD45+ immune cells were inhibited in the tumor tissues by mental stress exposure. FVS520 was used to distinguish living cells. Among the CD45+ cell population, tumor-associated macrophages (TAM) were further investigated using two markers F4/80 and CD163. As shown in Fig. 4C, D, mental stress exposure to the mice significantly increased the percentage of TAMs characterized by F4/80+ and CD163+ within tumors.

In order to further study the immune cells in tumors from the mice, paraffin sections were applied to stain T cells and TAMs in tumors. In consistent with the FACS analysis, mental stress exposure significantly reduced the amount of CD8+ T cells (Fig. 4E, F) while increased the amount of CD163+ TAMs (Fig. 4G, H).

### MiRNA-CCND1 signaling mediated the mental stress-induced cell cycle progression in tumor cells

**Fig. 5:**
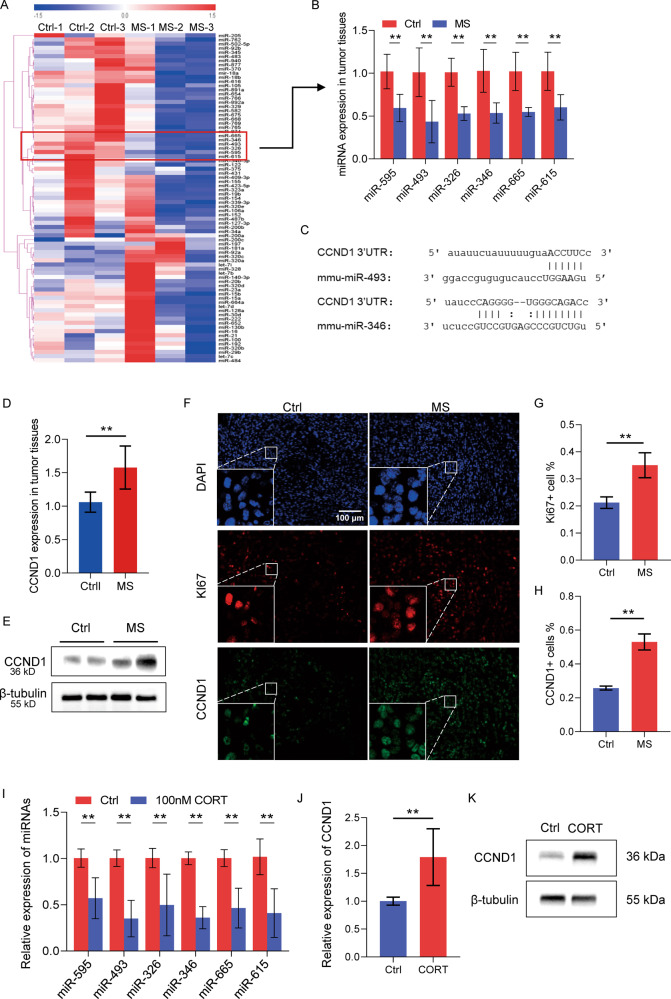
miRNA-CCND1 signaling mediated the mental stress-induced cell cycle progression in tumor cells. **A** Heatmap of the miRNA screening analysis of tumor samples from 3 stressed mice and 3 control mice, from which 6 downregulated miRNAs by mental stress were identified, including miR-326, 346, 493, 595, 615, and 665. **B** QRT-PCR validation of downregulation of miR-326, 346, 493, 595, 615, and 665 by stress stimulation. **C** Sequence blastn analysis showing the complementary binding interaction between CCND1 3′ UTR and miR-346/493. **D** QRT-PCR analysis showing the increased mRNA level of CCND1 in tumors of the stressed mice. **E** Western blot showing the increased protein level of CCND1 in tumors of stressed mice. **F** Immunofluorescence staining on the paraffin sections of tumors showing increased expression of both Ki67 and CCND1 in tumors of stressed mice. **G**, **H** Quantitative analysis of Ki67+ cells (**G**) and CCND1+ cells (**H**) in (**F**). **I** Doweregulation of miR-326, 346, 493, 595, 615, and 665 in 4T1 cells treated with corticosterone. **J**, **K** Upregulation of CCND1 at the levels of mRNA (**J**) and protein (**K**) in 4T1 cells treated with corticosterone. Data are presented as the mean ± SEM (*n* = 4 for Ctrl group, *n* = 5 for MS group). **p < 0.01.

To determine the miRNAs involvement in the stress-regulated tumor cell proliferation, miRNA screening analyses were performed in the tumor samples from three stressed mice and three control mice (Fig. 5A). Six miRNAs including miR-326, 346, 493, 595, 615, and 665 were identified with significant downregulation in tumors of the mice exposed to mental stress, compared to the three control mice (Fig. 5A). This was further validated by using quantitative real-time PCR analysis of the tumors (Fig. 5B). Target gene prediction analysis identified 298 genes as potential target genes of both miR-346 and miR-493, the top two most significantly downregulated miRNAs by stress stimulation (Fig. 5B, Supplemental Fig. S2). Gene ontology (GO) terms and Kyoto Encyclopedia of Genes and Genomes (KEGG) pathways analyses were subsequently performed on the 298 genes, indicating their involvement in the regulation of neuron system, cancer and other signaling (Supplemental Fig. S3A, B). These results strongly suggest the important function of miRNA-mRNA signaling in mediating the stress-induced cancer progression. Notably, as one of the 298 predicted target genes, CCND1 is a key regulator of the cell cycle. The sequence binding interactions between miR-346/493 and conserved 3′UTR of CCND1 mRNA were indicated in Fig. 5C. In consistent with the downregulation of miR-346/493, CCND1 showed significant upregulation at both mRNA and protein levels in tumors from the stressed mice (Fig. 5D, E). Analyses on the paraffin-embedded tissue sections further validated the stress-induced CCND1 expression and Ki67 expression in the mammary gland tumors (Fig. 5F–H).

In order to confirm the miRNA-CCND1 signaling regulation by mental stress exposure, exogenous CORT was used to treat 4T1 cells in vitro to mimic the stress hormone action on tumor cells in vivo. The cell cycle analysis demonstrated the induction of the S phase by CORT treatment (Supplemental Fig. S4A, B). Gene expression analysis validated downregulation of the six miRNAs in CORT-treated 4T1 cells (Fig. 5I), which was associated with upregulation of CCND1 at both mRNA levels (Fig. 5J) and protein levels (Fig. 5K).

## Discussion

About one fourth of the patients with breast cancer suffer substantial psychological distress including anxiety or depression [24, 25], which belongs to mental stress. Long-term or excessive mental stress exposure contributes to the high mortality and poor survival of cancer patients [26], mainly due to the significant influence on the nervous system, hormone secretion, endocrine balance, immune function and cellular metabolism [7, 27]. Although immune mechanisms and nonimmune mechanisms have been demonstrated to be responsible for the stress-induced healthy problem and diseases, the non-coding genome involvement in the stress-induced breast cancer regulation remains unclear. The current study applied a two-week unpredictable stress stimulation to the breast cancer tumor-bearing mice to mimic the patients suffering mental stress. Subsequent mechanism study not only validated the immune suppression, but also for the first time found the density induction of breast tumors by mental stress exposure. More importantly, this study revealed a non-coding miRNA-cell cycle signaling in mediating the stress-induced breast tumor cell proliferation and breast tumor growth in vivo.

Although cancer regulation by miRNAs has been widely studied, emerging evidence suggests a crosstalk between miRNAs and tumor microenvironment (TME) [28, 29]. MiRNAs not only regulate immune surveillance and immune response by targeting immune checkpoint inhibitors in TME [29], but also involve in the interactions between cancer cells and cancer-associated fibroblasts or cancer-related immune cells [28]. Our previous work also showed a miRNA cluster regulating cellular secretion of cytokines to control migration and invasion of neighboring cells in breast cancer through a heterotypic signaling in TME [30]. On the other hand, TME was demonstrated to play important roles in regulating miRNA biogenesis, methylation, and stability in cancer cells [28]. In consistence, the current study identified a miRNA-cell cycle signaling induced by stress hormones in regulating tumor growth.

Increased secretion of stress hormone, such as adrenocorticotropic hormone (ACTH), cortisol or CORT, activates the stress response pathways and triggers various biological processes to deal with stressful situations. It has been well confirmed that cortisol is the predominant corticosteroid stress hormone in human, while it is CORT in rats and mice. Literature has demonstrated the immunosuppressive and oncogenic function of stress hormones by accelerating cancer progression [31]. In consistence with the literature, the current study validated the increased levels of CORT in serum of the stressed mice, and also demonstrated a significant reduction of T cells and NK cells in both peripheral blood and tumor tissues by mental stress exposure. Moreover, the addition of exogenous CORT to the cell culture medium of 4T1 breast cancer cells mimicked the stress-induced phenotypes, including the promoted tumor cell proliferation and the accelerated cell cycle. These findings may suggest an adjuvant therapeutic potential of stress hormone inhibitor(s) to treat the cancer patients experiencing mental stress.

Estrogen and progesterone are two important hormones in women regulating the maintenance of female characteristics including mammary development, menstrual cycle and pregnancy. Estrogen and progesterone also promote the growth of luminal type of breast cancer. This subtype of breast cancer cells has expression of estrogen receptor (ER) and/or progesterone receptor (PR), thereby are sensitive to hormone therapy. Since the cancer cell 4T1 we used in the current study belongs to triple-negative breast cancer subtype lacking the expression of ER, PR and Her 2, we did not measure the change of estrogen and progesterone levels after exposure to mental stress. Whether these hormones have relationships with stress-induced phenotypes in the luminal type of breast cancer is yet to be determined. In addition, the current study demonstrated a long-lasting effect of mental stress on the tumor cells from mice. Even two weeks after the last stress stimulation, the primary tumor cells isolated from the stressed mice still maintained a higher cell proliferation rate than that from the control mice, suggesting a direct action of mental stress exposure and stress hormones on the tumor cells. These findings will be helpful to develop a new therapeutic strategy for cancer patients suffering mental stress.

In summary, the current study proved the increased stress hormone levels and promoted breast tumor growth by a two-week mental stress stimulation to mice. Stress-induced immune suppression was characterized by a significant reduction of T cells and NK cells, and induction of TAMs. Stress-induced tumor cell proliferation was at least partly due to the downregulation of miR-346, miR-493 and four other miRNAs and upregulation of CCND1 in tumor cells. These results not only demonstrated immune and nonimmune mechanisms for breast cancer progression induction by mental stress, but also revealed the involvement of non-coding genome in stress-regulated cell proliferation in breast cancer.

## Materials and methods

### Animals

6-week-old female BALB/c mice were purchased from Silaike Animal Company (Shanghai, China). All procedures on the animals were approved by the Institutional Animal Care and Use Committee of the Tongji University School of Medicine.

### Cell line and cell culture

Breast cancer cell line 4T1 was originally obtained from American Type Culture Collection (ATCC), maintained in our lab and passed the test for mycoplasma contamination. The cells were cultured at 37 °C with 5% CO_2_ in Roswell Park Memorial Institute-1640 (RPMI-1640) medium supplemented with 10% fetal bovine serum and 1% penicillin–streptomycin.

### Preparation of cell transplantation-derived-xenograft model of breast cancer

4 × 10^4^ 4T1 cells in 100 µl PBS were mixed with Matrigel and injected into the fourth fatty pad of the mice (*n* = 10). They were randomly separated into two groups (*n* = 5 in each group) by using an on-line tool (www.random-online.com). One group of mice lived normally without any stimulation (Ctrl group), while the other group of mice received mental stress stimulation (MS group) (special note: Accident death happened to one of the five control mice in the middle of the study due to a reason unrelated to the experiments. So final *n* = 4 for the control group). Tumor volumes were measured every 5 days until day 28 after cell transplantation when all the mice were sacrificed. No blinding was applied to the experiments.

### Mental stress stimulation

The mice in the MS group received stress stimulations between 7 am and 7 pm every day for continuous two weeks. The unpredictable stress stimuli include noise stress (80 decibels for 30 min), restraint stress (30 min), elevated platform stress (10 cm diameter, 1.2 m height of platform for 15 min), shaking stress (60 rpm for 15 min), and somatosensory stress (tail-clamping for 1 min, repeat 3 times in 10 min). Each mouse was unpredictably given two of the six stimuli at a random selection every day.

### Animal behavior tests

All the mice received three kinds of behavior tests within the two weeks after the last stress stimulation, including the open field test, the tail suspension test and the sucrose preference test, following the procedures described in the literature [32, 33]. A video tracking system (SuperMaze V2.0, Shanghai, China) was used to record and analyze the activity of mice in the open field test and tail suspension test.

### Quantitative analysis of miRNAs

A home-made QRT-PCR-based miRNA panel containing 88 cancer-related human miRNAs was applied for miRNA screening analysis. 500 ng of total RNA was used for first strand cDNA synthesis by using the M&G miRNA Reverse Transcription kit (miRGenes, China) following the manufacturer’s instruction. SYBR Green Master Mix (2Χ, Applied Biosystem, USA) and QuantStudio™ 6 Flex Real-Time PCR System (Applied Biosystem, USA) were used for real-time PCR analysis. 5s ribosome RNA was used for normalization. The sequences of all the tested miRNAs are available upon request. Primers were synthesized by GenScript (Nanjing, China).

### Flow cytometry

For peripheral blood samples, RBC lysis buffer (BioLegend, USA) was used to remove the red blood cells following the manufacturer’s instruction. For tumor tissue samples, collagenase I (150 U/ml), hyaluronidase (100 U/ml), and DNase I (0.33 U/ml) were applied to digest the tissues into single-cell suspension. Fluorescent-conjugated antibodies were applied for staining for 25 min at 4 °C in the dark, including CD45-PE-Cy7 (30-F11), CD3-FITC (17A2), CD4-PE (GK1.5), CD8-perpcCY5.5 (53-6.7), CD49b-APC (DX5), CD19-PE-Cy7 (1D3) and F4/80-PE (T45-2342) from BD Pharmingen (San José, USA), and CD163-APC (S15049I) from BioLegend (San Diego, USA). FACSAria II (BD, USA) was used for analysis.

### Immunohistochemistry (IHC) and immunofluorescence (IF) staining

Tumor samples were fixed in 4% paraformaldehyde (Beyotime, China) and embedded in paraffin. The slides were incubated with primary antibodies at 4 °C overnight, then with HRP-conjugated goat anti-rabbit IgG secondary antibody (for IHC) or fluorescent-conjugated secondary antibody (for IF) at room temperature for 1 h in dark. The primary antibodies include: Ki67 (ab16667, Abcam), CD163 (ab182422, Abcam), CD4 (ab183685, Abcam), CD8 (ab217344, Abcam) and CCND1 (55506, Cell Signaling Technology). HRP-linked goat anti-rabbit IgG (ab6721, Abcam), Alexa Fluor^®^ 488-linked goat anti-rabbit IgG (ab150077, Abcam) and Alexa Fluor^®^ 555-linked goat anti-rabbit IgG (ab150078, Abcam) were used as secondary antibodies. Leica DM300 microscope was used to capture images. Leica Application Suite X software 3.0.0.15697 was used for quantitative analysis.

### MiRNA-target prediction and pathway analysis

Target gene prediction of miRNAs was performed using the Encyclopedia of RNA Interactomes (ENCORI, http://starbase.sysu.edu.cn/index.php) [34]. GO and KEGG pathway analyses were performed using WEB-based Gene SeT AnaLysis Toolkit (WebGestalt, http://www.webgestalt.org/) [35].

### Statistical analysis

Data are presented as mean ± SEM unless otherwise stated. All experiments were repeated at least three times independently with triplicates. Two-tailed *t* test was used to analyzing the independent samples between groups. All the data met normal distribution requirement of tests. *P* < 0.05 was considered as statistical significance.

## Supplementary information

Supplemental information

## Data Availability

The original data presented in this study are included in the article/supplementary information. Further inquiries are available upon request to the corresponding author.
