# Supplementary material for: Immune and nonimmune mechanisms mediate the mental stress-induced tumor growth in a xenograft model of breast cancer
Source: Cell Death Dis. 2021 Oct 23;12(11):987. doi: 10.1038/s41419-021-04280-9 (PMC8542049; doi:10.1038/s41419-021-04280-9)

Supplemental information

Immune and nonimmune mechanisms mediate the mental stress-induced tumor growth in a xenograft model of breast cancer

Wenjing Ma[1#], Pengfei Liu[1#], Jie Zheng[2#], Jinhui Lü[1], Qian Zhao[1], Danni Li[1], Yuefan Guo[1], Lu Qian[1,3], Qiong Wang[1], Xinman Miao[1,3] and Zuoren Yu[1*]

[1]Research Center for Translational Medicine, Shanghai East Hospital, Tongji University School of Medicine, Shanghai, China.
[2]School of Life Sciences and Technology, Tongji University, Shanghai, China.
[3]Jinzhou Medical University, School of Basic Medical Sciences, Jinzhou, Liaoning Province, China.

Supplemental Figure Legends

Supplemental Figure S1. Dynamic change of the body weight of the mice in both MS and Ctrl groups. Data are presented as the mean ± SEM (n=4 for Ctrl group, n=5 for MS group).

Supplemental Figure S2. miRNA target gene prediction analysis derived 298 genes potentially targeted by both miR-346 and miR-493 in rodents, including CCND1.

Supplemental Figure S3. GO terms (A) and KEGG pathways (B) analysis of the 298 target genes of miR-346 and miR-493 showing most significant pathways including neuron regulation, cell apoptosis & death regulation and cancer-related regulation.

Supplemental Figure S4. A. The cell cycle analysis of 4T1 cells with or without treatment of corticosterone. B. Quantitative analysis of the cells at different phases in A. Data are presented as the mean ± SEM (n=3). **$p<0.01$.

Supplemental Figure S1

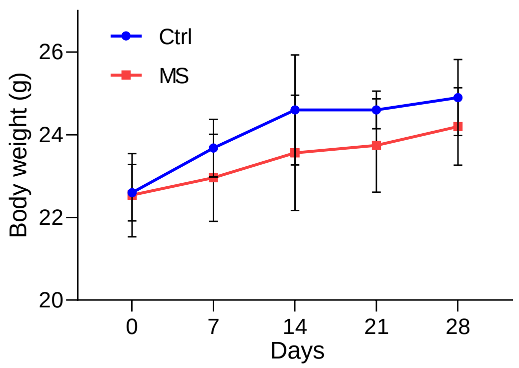

Supplemental Figure S2

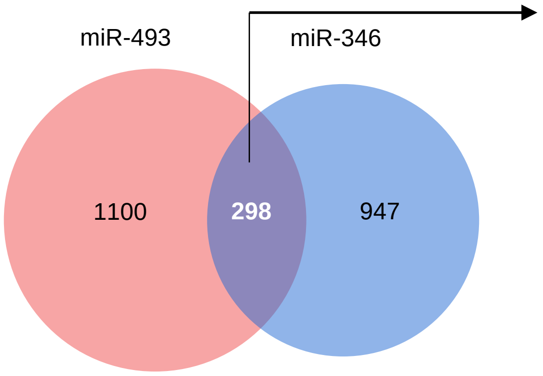

**Ccnd1**	Tmbim6	Pfkfb1	Abca5	Surf4	Fam168a	Purb
Ncoa2	Scn8a	Snx30	Nploc4	Stom	Bag3	Rmnd5b
Fam168b	Pak2	Nfia	Csnk1d	Zeb2	Cd151	Ndel1
Pgap1	Ubn1	Cc2d1b	Adcy1	Madd	Tspan4	Nlgn2
Kif1a	Yeats2	Foxj3	Sertad2	Ldlrad3	Eri1	Akap1
Steap3	Opa1	Grik3	Pank3	Nlgn3	Asah1	Kpnb1
Btg2	Fstl1	Phc2	Aff4	Rasgrp1	Stox2	Pitpnc1
Lamc1	Qk	Fndc5	G3bp1	Dzank1	Ncan	Cfl1
Ier5	Pdpk1	Yars	Tsr1	Zfp120	Crtc1	Fads1
Mpzl1	Fmr1	Ece1	Crk	Plagl2	Nr2f6	Osbp
Pea15a	Ubr2	Rere	Flot2	Blcap	Slc35e1	Gnaq
Akt3	Foxp4	Ccnl2	Zfp652	Znfx1	Smim7	Btaf1
Inpp4a	Khsrp	Adam22	Mllt6	Spata2	Cmtm4	Nacc2
Nemp2	Rab31	Cplx1	Dcaf7	Tmem189	Il34	Sec16a
Pikfyve	Memo1	Ulk1	Egln3	Nfatc2	Ikbkg	Furin
Ctdsp1	Fam98a	Kctd10	Tmem229b	Zfp512b	Kat6a	Homer2
Eif4e2	Rhoq	Ncor2	Asxl2	Ncs1	Sfrp1	Arnt2
Capn10	Socs5	Eif4h	Rock2	Dab2ip	Erlin2	Xpo6
Zcchc2	Aqp4	Rac1	Pacs2	Kif5c	Tusc3	Clip3
Tor1aip2	Nr3c1	Smurf1	E2f3	Chp1	Wdr83os	Igf1r
Abl2	Rnf165	Pcgf3	Slc22a23	Stk35	Nfat5	Rgma
Desi2	Impact	Sppl3	Golm1	Itch	Zfhx3	Dap
Kcnh1	Map3k2	Med13l	Scamp1	Epb41l1	Galnt2	Sema5a
Utrn	Pcdha11	Bcl7a	Map3k1	Ss18l1	Gm20388	Zfp41
Ccdc28a	Pcdha3	Fam20c	Klf6	Dnajc5	Fam118b	Gtpbp1
Tnfaip3	Pcdhac2	Trrap	Prpf4b	Zfp704	Arhgef12	Tnrc6b
Col6a1	Pcdha5	Cpsf4	Rreb1	Gnb4	Tcf12	Zc3h7b
Rnf126	Pcdhac1	Plxna4	Nsd1	Setd7	Ppm1m	Ano6
Ap3d1	Pcdha12	Vopp1	Ajuba	Kpna4	Trim71	Rnf38
Slc39a3	Pcdha1	Zfp9	Extl3	Gria2	Pin1	Smap2
Nfic	Pcdha6	Mical3	Dpysl2	Ahcyl1	Ilf3	Zfp362
Gas2l3	Pcdha9	Tspan9	Samd8	Ank2	Nectin1	Tnfrsf1b
Nab2	Pcdha8	Kras	Il17rd	Srsf11	Rdx	Kif1b
Lrp11	Pcdha2	Peg10	6-Mar	Zc2hc1a	Sema7a	Tgfbr1
Fuca2	Pcdha4	Aak1	Phf5a	Pcdh10	Wdr82	Ammecr1
Ccdc6	Pcdha7	Slc6a6	Pacsin2	Mbnl1	Cdc25a	D17Wsu92e
Midn	Eif1a	Bhlhe40	Ttll12	Mfsd1	Ubp1	E130308A19Rik
Zbtb7a	Nedd4l	Arl8b	Hdac7	Etv3	Cmtm6	D430041D05Rik
Timp3	Mbp	Rasgef1a	Arf3	Pbxip1	Dynlt3	2310022B05Rik
Slc6a15	Capn1	Foxj2	Kmt2d	Mib2	Ids	4933434E20Rik
E2f7	Ranbp6	Shisa7	Itga5	Txnip	Zfx	
Ankrd52	Mgea5	Ehd2	Prkaa1	Sec24d	Zmym3	
Nudcd3	Fosl1	Capns1	Lifr	Fubp1	Rlim	

Supplemental Figure S3

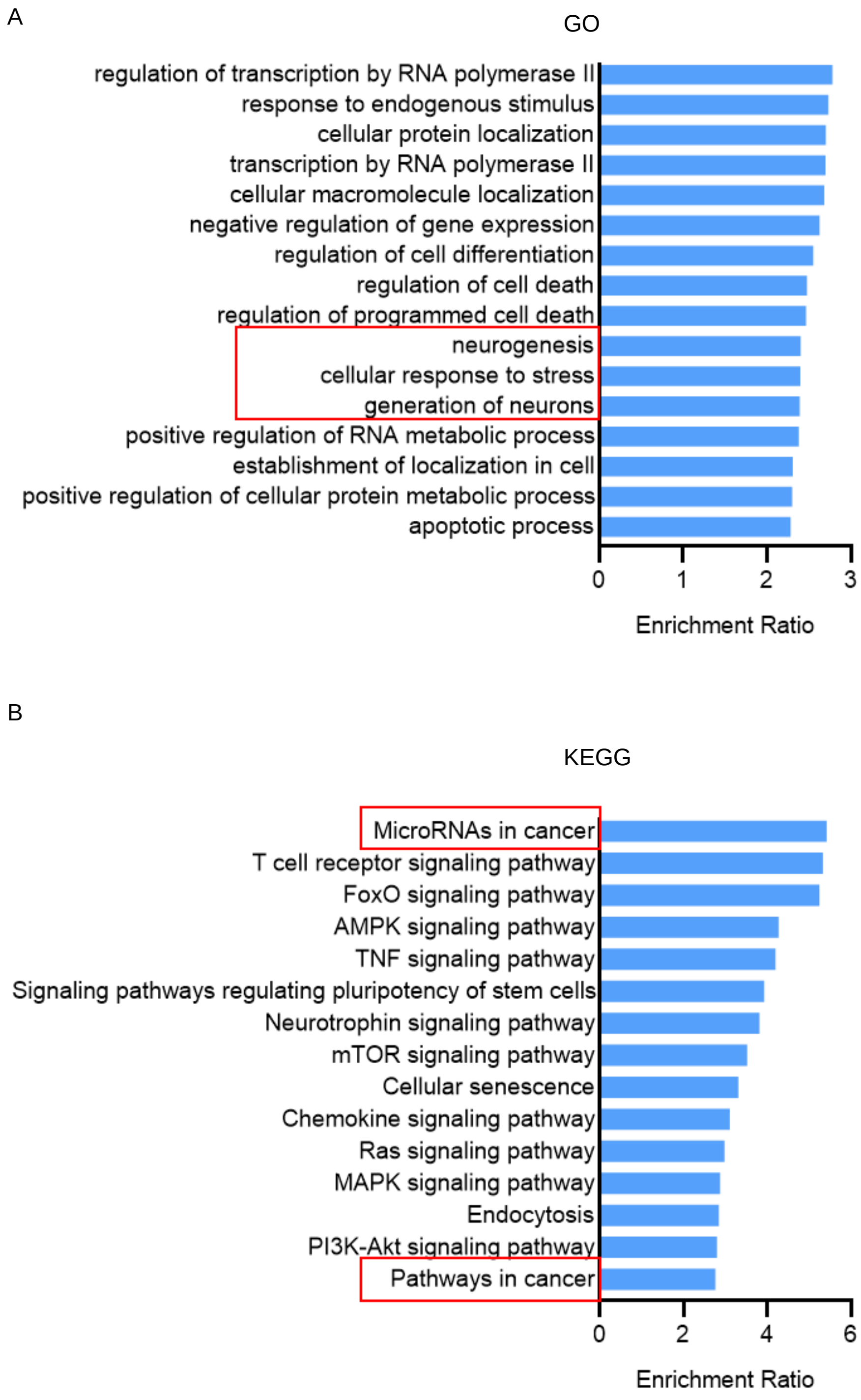

Supplemental Figure S4

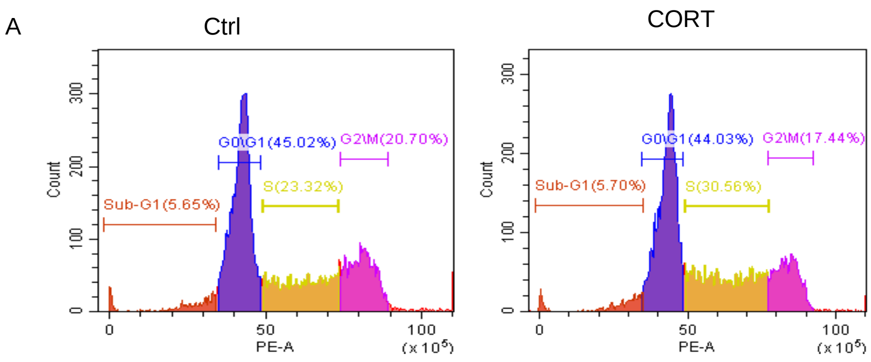

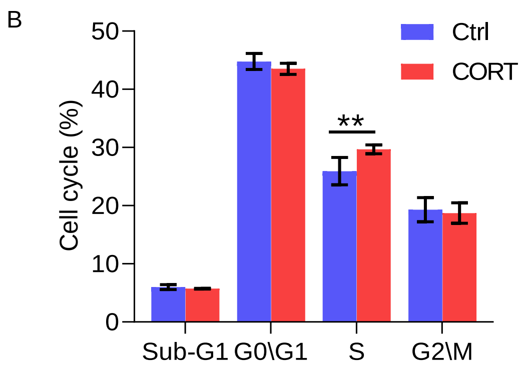

Supplement: Supplementary file 1 — Supplemental information [file 41419_2021_4280_MOESM1_ESM.pdf]
